# Elaiophylin Is a Potent Hsp90/ Cdc37 Protein Interface Inhibitor with K-Ras Nanocluster Selectivity

**DOI:** 10.3390/biom11060836

**Published:** 2021-06-04

**Authors:** Farid A. Siddiqui, Vladimir Vukic, Tiina A. Salminen, Daniel Abankwa

**Affiliations:** 1 Turku Bioscience Centre, University of Turku and Åbo Akademi University, 20520 Turku, Finland; farid.siddiqui@utu.fi (F.A.S.); vukicv@uns.ac.rs (V.V.); 2 Faculty of Technology, University of Novi Sad, 21000 Novi Sad, Serbia; 3 Structural Bioinformatics Laboratory, Biochemistry, Faculty of Science and Engineering, Åbo Akademi University, 20520 Turku, Finland; Tiina.Salminen@abo.fi; 4 InFLAMES Research Flagship Center, Åbo Akademi University, 20520 Turku, Finland; 5 Cancer Cell Biology and Drug Discovery Group, Department of Life Sciences and Medicine, University of Luxembourg, 4362 Esch-sur-Alzette, Luxembourg

**Keywords:** K-Ras, Hsp90, Cdc37, nanoclustering, cancer, drug development

## Abstract

The natural product elaiophylin is a macrodiolide with a broad range of biological activities. However, no direct target of elaiophylin in eukaryotes has been described so far, which hinders a systematic explanation of its astonishing activity range. We recently showed that the related conglobatin A, a protein–protein interface inhibitor of the interaction between the N-terminus of Hsp90 and its cochaperone Cdc37, blocks cancer stem cell properties by selectively inhibiting K-Ras4B but not H-Ras. Here, we elaborated that elaiophylin likewise disrupts the Hsp90/ Cdc37 interaction, without affecting the ATP-pocket of Hsp90. Similarly to conglobatin A, elaiophylin decreased expression levels of the Hsp90 client HIF1α, a transcription factor with various downstream targets, including galectin-3. Galectin-3 is a nanocluster scaffold of K-Ras, which explains the K-Ras selectivity of Hsp90 inhibitors. In agreement with this K-Ras targeting and the potent effect on other Hsp90 clients, we observed with elaiophylin treatment a submicromolar IC_50_ for MDA-MB-231 and MIA-PaCa-2 3D spheroid formation. Finally, a strong inhibition of MDA-MB-231 cells grown in the chorioallantoic membrane (CAM) microtumor model was determined. These results suggest that several other macrodiolides may have the Hsp90/ Cdc37 interface as a target site.

## 1. Introduction

The C_2_-symmetric 16-member macrodiolide elaiophylin was isolated from *Streptomyces melanosporus*, an actinomycete strain, in 1959 as azalomycin [1]. It is produced by various Streptomyces species and at least 19 natural derivatives of elaiophylin are known [1]. Biosynthesis of elaiophylin occurs on modular polyketide synthase assembly lines, similar to that of other macrodiolides like conglobatins [2,3].

Elaiophylins have an astonishingly broad spectrum of biological activities, including antibiotic, antiviral, antifungal, antiprotozoan, anthelmintic, immunosuppressive, and anticancer activities [1,4,5,6]. While their antibacterial activity might be attributed to their ionophoric activity, i.e., they are able to form cation-selective ion channels in membranes [7], their mechanism of action in eukaryotes is likely different and may include several targets.

Several cancer cell lines, including A549, PC3, and MCF-7 cells, respond in the low micromolar range to close derivatives of elaiophylin [4,8]. Elaiophylin itself has a potent anti-cancer activity, which as a single agent (2 mg/ kg q.a.d) can reduce tumor growth and metastization in a SKOV3-cell-line-derived orthotopic ovarian cancer model, without notable toxicities [6]. Treatment of SKOV3 cells was found to inhibit late stages of autophagy, similarly to the established inhibitor chloroquine, thus blocking autophagic flux and inducing cell death. A similar activity was seen in TP53 mutant U266 multiple myeloma cells in mouse xenografts [9].

Elaiophylins belong to the group of macrodiolides, specifically macrocyclic lactone natural products, which also include conglobatins. Conglobatin A is known as Hsp90/ Cdc37 protein–protein interface inhibitor [10]. The widely expressed major protein folding chaperone Hsp90 employs additional co-chaperones, such as Cdc37, which delivers kinase clients to Hsp90 [11,12]. We previously identified conglobatin A to have K-Ras-directed anti-cancer stemness activity [13,14]. Recently, we confirmed that conglobatin A is a protein–protein interface inhibitor, interfering with the binding of Cdc37 to the N-terminus of Hsp90 and delineating the K-Ras-dependent mechanistic background of this activity [15]. Conglobatin A treatment destabilizes the Hsp90 client HIF1α and downregulates its transcriptional target galectin-3 (Gal3) [15]. Gal3 is a well-established nanocluster scaffold of K-Ras4B (hereafter K-Ras), i.e., it stabilizes oligomeric MAPK-signaling complexes of K-Ras in the plasma membrane, thus promoting its activation [16,17,18]. Analogously to the related galectin-1, nanocluster stabilization by Gal3 may occur by increasing the complexation time between Ras and the effector Raf [19,20]. Importantly, this K-Ras-directed activity was also observed with the Hsp90 ATP-pocket inhibitor 17-AAG (17-N-allyloamino-17-demethoxygeldanamycin), suggesting that Hsp90 inhibitors may have, in general, interesting potential in KRAS mutant cancers. Importantly, based on our mechanistic biomarkers HIF1α and Gal3, we predicted that pancreatic cancers are particularly well amenable to Hsp90 inhibitor treatment, as both of these biomarkers are highly expressed in that cancer type [15].

While in the past Hsp90 inhibitor development focused on ATP-pocket competitive inhibitors, alternative targeting strategies, such as interference with the Hsp90/ Cdc37 interaction are gaining track [21,22]. However, protein interfaces are notoriously difficult to target and require special screening strategies for inhibitor identification. We recently implemented a split-Luciferase-based assay to identify Hsp90/ Cdc37 interaction inhibitors [23]. This assay allowed us to profile Hsp90 inhibitors with different mechanisms of action, confirming, for instance, Withaferin A as Hsp90/ Cdc37 interaction inhibitor of putative allosteric C-terminal-type, while devalidating platycodin D as a putative direct-interface-inhibitor-type Hsp90/ Cdc37 inhibitor [23].

Only recently has the group of Quidong You managed to develop mature and potent (K_d_ = 0.5 µM) Hsp90/ Cdc37 interface inhibitors with anti-tumor activities in vivo [22,24]. Macrocycles such as elaiophylin and conglobatin A may, in general, be attractive as interface inhibitors, given that they can cover a larger surface area. They may therefore serve as interesting starting points for protein interface inhibitor development. Indeed, based on conglobatin A as a pharmacophore, we recently identified by a combination of computational and biochemical screening of >7 M compounds, six new small molecule Hsp90/ Cdc37 protein interface inhibitors with low micromolar activity [15]. These hits could serve as starting points for novel, more potent small molecule Hsp90/ Cdc37 interface inhibitors.

Here, we established that elaiophylin has an even more potent Hsp90-inhibitory activity than conglobatin A. Elaiophylin interferes with the interaction of Cdc37 and the N-terminus of Hsp90, and depletes Gal3, thus selectively decreasing K-Ras, but not H-Ras, nanoclustering. This activity is shared with conglobatin B1 and C1, supporting the notion that macrodiolides may have a promising potential as protein–protein interface inhibitors of Hsp90/ Cdc37. Finally, we demonstrated that elaiophylin potently decreases 3D sphere formation of MDA-MB-231 cells with submicromolar IC50 and microtumor growth of the same cells in the chorioallantoic membrane (CAM) model.

## 2. Material and Methods

### 2.1. DNA Constructs, Compounds, Antibodies, and Reagents

pcDNA3.1 (+)-NRL-Hsp90, pcDNA3.1 (+)-NRL-N-Hsp90, and pcDNA3.1 (+)-Cdc37-CRL have been previously described [15,23]. Plasmids pmGFP-/ mCherry-K-RasG12V, and pmGFP-/ mCherry-H-RasG12V have been previously described [25,26]. DMEM (cat. no. D617), RPMI (cat. no. R586), L-glutamine (cat. no. G7513), and Mowiol 4-88 (cat. no. 813819) were purchased from Sigma-Aldrich (St. Louis, MO, USA). Fetal bovine serum (cat. no. S1810) was obtained from Biowest. Transfection reagent, jetPRIME (cat. no. 114-75) was purchased from Polyplus. RIPA buffer (cat no. 89900), protease, and phosphatase inhibitors (cat. no. A32959) were obtained from ThermoFisher Scientific (Waltham, MA, USA). Conglobatin A (cat. BIA-C1022), conglobatin B1 (cat. BIA-C1980), conglobatin C1 (cat. BIA-C2682), and elaiophylin (cat no BIA-E1028) were purchased from Bioaustralis. 17-AAG (cat. sc-200641) was purchased from Santa Cruz Biotechnology. (Dallas, TX, USA). All compounds were dissolved at a stock concentration of 10 to 20 mM in DMSO (cat. No. BP-231-100, Fisher Scientific). Primary antibodies against Hsp70 (cat. no. MA3-006) were purchased from ThermoFisher Scientific; C-Raf (cat. no. sc-133), galectin-3 (cat. no. sc-20157), Hsp90 (cat. no. sc-69703), and Cdc37 (cat. no. sc-13129) were obtained from Santa Cruz Biotechnology; Akt (cat. no. 9272S) and ERK1/2 (cat. no. 9102S) from Cell Signaling Technology; HIF-1α (cat. no. NB-100-134) from Novus Biologicals and β-actin (cat. no. A1978) was purchased from Sigma-Aldrich. As secondary antibodies, we employed HRP-conjugated anti-mouse (cat. no. sc-2954, Santa Cruz Biotechnology) and anti-rabbit antibodies (cat. no. HAF008, R&D System).

### 2.2. Cell Culture

HEK293 and MIA PaCa-2 were cultured in Dulbecco’s modified Eagle’s medium (DMEM) containing 10% fetal bovine serum and 2 mM L-glutamine. MDA-MB-231 was cultured in RPMI containing 10% FBS and 2 mM L-glutamine. All cell lines were subcultured twice a week and incubated at 37 °C with 5% CO_2_ in a humidified cell incubator.

### 2.3. Fluorescence Lifetime Imaging Microscopy (FLIM)-FRET

HEK cells were seeded in a 12-well plate onto sterile 16 mm coverslips in complete 1 mL DMEM media. The next day, cells were transfected with 500 ng mGFP-K-RasG12V or mGFP-H-RasG12V for donor fluorophore expression using jetPRIME transfection reagent. For the nanoclustering-FRET pair expression, cells were co-transfected with mGFP-K-RasG12V and mCherry-K-RasG12V or mGFP-H-RasG12V and mCherry-H-RasG12V at a ratio of 1:3 and total of 800 ng plasmids. The next day, cells were treated with 0.1% DMSO control or indicated inhibitors. After 24 h of treatment cells were fixed in 4% paraformaldehyde for 10 min. Cells were mounted with Mowiol after washing with PBS. A fluorescence microscope (Zeiss AXIO Observer D1) equipped with a fluorescence lifetime imaging attachment (Lambert Instruments) was used to measure the fluorescence lifetime of the donor fluorophore mGFP. The apparent FRET efficiency (E_app_) percentage was calculated using the formula: E_app_ = (1 − τ_DA_/τ_D_) × 100%, where τ_DA_ is the lifetime of the donor fluorophore in the presence of acceptor and τ_D_ is the lifetime of donor fluorophore only [25,27].

### 2.4. Western Blotting

Cells were lysed in RIPA buffer containing protease and phosphatase inhibitor cocktails, along with 1 mM phenylmethylsulfonyl fluoride. After addition of lysis buffer, cells were placed on ice for 20 min and then sonicate for 5 min. Cell lysates were centrifuged at 13,000× *g* for 10 min at 4 °C. Protein was quantified using BCA kit (cat. no. 23225, ThermoFisher Scientific). Next, 10 µg protein samples were separated on 10% SDS PAGE gels. Proteins were transferred onto nitrocellulose membrane (cat. no. NBA 083C001EA, Protran) using a wet transfer system or semidry Transferblot Turbo Transfer System (Bio-Rad, Hercules, CA, USA). The transferred proteins were membrane blocked in 5% skimmed milk for 30 min. The membrane was incubated with primary antibody at a 1:2000 dilution in 5% BSA overnight at 4 °C. The next day, membranes were washed for 3 × 10 min and then incubated with HRP-conjugated secondary antibody for 1 h at room temperature. After the ECL-reaction (cat. no. 170-5061, BioRad), protein bands were quantified using a ChemiDoc MP instrument (Bio-Rad). All of the antigens and molecular weights in kDa are labelled next to the blots on the figures.

### 2.5. Hsp90/ Cdc37 Split Renilla Luciferase Assays

HEK cells were seeded in a 10 cm dish and transfected the next day with 10 µg plasmid pcDNA3.1(+)-NRL-Hsp90, pcDNA3.1(+)-NRL-N-Hsp90, or pcDNA3.1(+)-Cdc37-CRL using jetPRIME. After 48 h, cells were harvested and lysed in 1 mL 1 × lysis buffer provided in the *Renilla* Luciferase Reporter Assay System Kit from Promega (cat. E2820) and incubated on ice for 10 min. Cell lysates were then cleared by centrifugation at 13,000× *g* for 1 min. Then, 20 µL assay buffer from the aforementioned kit was added to each well of a white 96-well flat-bottom plate (cat. 3917, Costar, Corning Inc., Corning, NY, USA). After that, test compounds or 0.1% DMSO control, as indicated, were added to each well. Then, 5 µL NRL-Hsp90- or NRL-N-Hsp90-lysate was added to each well one row at a time. Then, the plate was incubated for 5 min at RT. After that, 5 µL Cdc37-CRL was added and incubated for 2 min at RT. Just before reading, 20 µL assay buffer containing 2 × *Renilla* luciferase substrate (coelenterazine) was added and the plate was read using a Synergy H1 Hybrid Multi-Mode reader (BioTek) in the luminescence detection mode, as described previously in detail [23].

### 2.6. Hsp90 N-Terminal ATP-Binding Site Competition Assay

The competitive, N-terminal Hsp90 ATP-pocket binding assay was performed by using the Hsp90α (N-terminal) Assay Kit (cat. no. 50298, BPS Bioscience, San Diego, CA, USA), according to the manufacturer’s instructions. The black, low-binding NUNC microtiter plate, included in the kit, was used and the total reaction volume was 50 μL. FITC-labeled geldanamycin (5 nM) was combined with test compounds in the kit assay buffer, 2 mM DTT (cat no. BP172, Thermo Fisher Scientific) and 100 µg/ mL BSA (cat. no. A8022, Sigma-Aldrich). After that, Hsp90α was added to each well and incubated at RT with slow shaking for 2 h. All samples were analyzed in triplicate. The fluorescence polarization of the samples was measured using a Synergy H1 Hybrid Multi-Mode reader (BioTek) with a polarization cube (excitation 485 ± 10 nm, emission 528 ± 10 nm), as described previously [15]. The fluorescence polarization was determined as $\mathrm{mP}\;=(\left(I_{\mathrm{II}}\;–{\;I}_{⊥}\right)/\left(\left(I_{\mathrm{II}}+{\;I}_{⊥}\right)\right)\times 1000$, where I_II_ is the intensity of the parallel and $I_{⊥}$ the intensity of the perpendicular polarized light.

### 2.7. Tumorosphere Assay (3D Spheroid Culture)

Here, 2000 MDA-MB-231 cells or 4000 MIA-PaCa-2 cells were seeded in 100 µL RPMI or DMEM medium, respectively, in flat bottom 96-well suspension culture plates (cat. no. 655185, Cellstar, Greiner Bio-One) containing 1× B27 (cat. no. 17504044, Gibco, Thermo Fisher Scientific), 25 ng/ mL EGF (cat. no. E9644, Sigma-Aldrich), and 25 ng/ mL FGF (cat. no. RP-8628, Thermo Fisher Scientific). Then, 25 µL medium was added every 2 days and cells were allowed to form spheres for 6 days. After 6 days, 50 µL medium containing 0.3% DMSO or 3 × of indicated compound concentrations were added to the wells. After 72 h of treatment, 10% (*v*/*v*) alamarBlue reagent (cat. no. DAL1100, Thermo Fisher Scientific) was added to each well. After 3 h, the fluorescence intensity was measured (excitation 560 nm ± 10 and emission 590 ± 10 nm) using a Synergy H1 Hybrid Multi-Mode reader (BioTek).

### 2.8. Cell Viability Assay (2D Cell Proliferation)

Here, 500 MDA-MB-231 or 1000 MIA-PaCa-2 cells were seeded in 96-well flat bottom culture plates in 100 µL medium. After 24 h, cells were treated with 0.1% DMSO vehicle control or elaiophylin in serial dilution in DMSO using a 1:2 dilution-series starting at 2 µM. After 3 days of treatment, 10% alamarBlue reagent per well was added. After 3 h of incubation under normal culture conditions, the fluorescence intensity (excitation 560 ± 10 nm and emission 590 ± 10 nm) per well was measured using a Synergy H1 Hybrid Multi-Mode reader (BioTek).

### 2.9. Chick Chorioallantoic Membrane (CAM) Microtumor Assay

Fertilized eggs were incubated at 37 °C in a 55% to 60% humidified egg hatcher incubator (MG100, Fiem). After 2 days, a small hole was made and covered using parafilm. After 8 days, the hole was enlarged and 2 million MDA-MB-231 cells were resuspended in 1 × PBS and Matrigel (cat. no. 356234, BD Biosciences, Franklin Lakes, NJ, USA) at a ratio of 1:1 and deposited within a sterilized 7 mm diameter plastic rings cut from a plastic pipette (cat. no. LW4111, Alpha Laboratories Limited), which was placed on the large blood vessel on the surface of the chicken embryo CAM. The next day, xenografted microtumors were treated with 20 µL 0.1% DMSO vehicle control or test compound diluted in 1 × PBS. Microtumors were treated once every day and collected after 5 days of treatment. Then, microtumors were transferred into pre-weighted 1.5 mL Eppendorf tubes containing 500 µL PBS and the tumor mass was determined by the difference of the weights.

### 2.10. Coimmunoprecipitation Experiments

One million MDA-MB-231 cells were seeded in a 10 cm dish in RPMI medium or two million MIA PaCa-2 cells seeded in a 10 cm dish in DMEM medium. The next day, cells were treated with 0.1% DMSO control or an indicated concentration of elaiophylin or conglobatin A. After 24 h of treatment, cells were lysed in the lysis buffer provided with the CO-IP kit (cat no. 90409) Thermo Fisher Scientific. Then, 800 µg cell lysate was incubated with 5 µL (0.5 µg) Hsp90 antibody overnight at 4 °C in a total of 500 µL lysis buffer. For the negative control, 800 µg DMSO treated cell lysate was incubated with 0.5 µg mouse IgG isotype control (cat. no. 10400C, Thermo Fisher Scientific). The next day, antigen–antibody complexes were transferred to a tube containing 25 µL protein A/G magnetic beads and incubated for 2 h at 4 °C with mixing. Magnetic-bead-bound proteins were eluted by using the manufacturer’s instructions in 100 µL elution buffer. Finally, 20% of eluted immunoprecipitated proteins were analyzed using Western blotting.

### 2.11. Computational Docking Study

The 3D structure for elaiophylin was constructed using Maestro software in the Schrödinger package (Maestro, 2020, **Schrödinger Release 2020-4:** Schrödinger, LLC, New York, NY, USA, 2020). The geometry optimization was done using the OPLS3 force field and the Powell conjugated gradient algorithm method with convergence criterion of 0.01 kcal/ [mol Å] and the maximum iterations set of 1000 [28].

The three-dimensional structure for HSP90 was retrieved from the Protein Data Bank (PDB ID: 3T0Z). All protein simulations were performed using the OPLS3 force field [28]. The protein structure was prepared for simulations using Protein Preparation Wizard in Schrödinger—the hydrogen atoms were added, the protonation types were solved using Epik (pH: 7.0 ± 2.0), the bond orders were assigned, and the water molecules were removed [29]. The potential binding site of elaiophylin was determined by utilizing the crystal structure of yeast N-Hsp90 in combination with the C-terminal domain of human Cdc37 (PDB ID 1US7) [30], as well as the predicted binding site of conglobatin A [15]. The molecular docking simulation was performed with Glide using the extra precision (XP) mode with ligand flexibility and the Epik state penalties for the docking score [31]. The MM-GBSA free energy for XP docked binding pose of Elaiophylin was calculated using the VSGB 2.0 solvation model, allowing flexibility for the residues within 6.0 Å distance from the docked compound [32]. Visualization of the obtained results was done using PyMOL (The PyMOL Molecular Graphics System, Version 2.0 Schrödinger, LLC).

### 2.12. Statistical Analysis

GraphPad Prism software (version 6) was used for the statistical analysis. The sample sizes of independent biological repeats, *n*, for each data set are given in the figure legends. All of the in text citations and graphs show mean values ± SEM across all of the technical and biological repeats. Unless otherwise stated, we employed one-way ANOVA with Tukey’s multiple comparison test or Student’s test to determine the statistical differences to the control samples. A *p*-value of <0.05 is considered statistically significant. The statistical significance levels are annotated in the plots as * *p* < 0.05; ** *p* < 0.01; *** *p* < 0.001; **** *p* < 0.0001.

## 3. Results

### 3.1. Elaiophylin Inhibits the Hsp90/ Cdc37 Complex Formation

**Figure 1:**
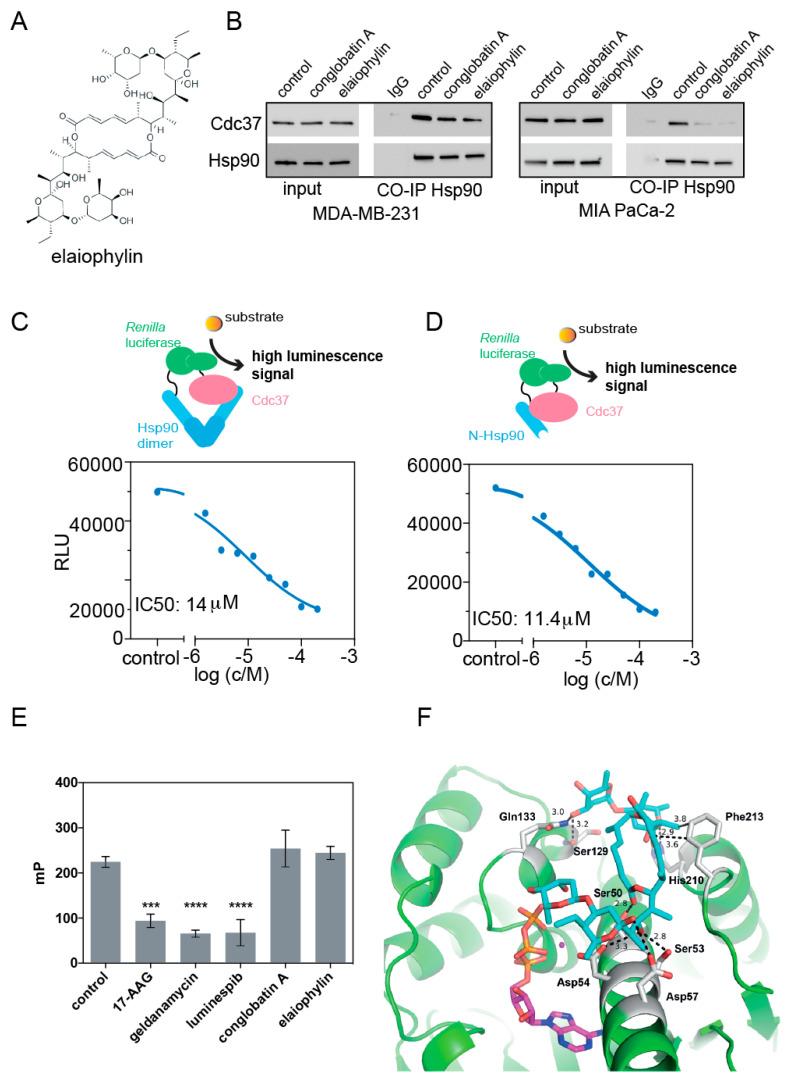
Elaiophylin disrupts the interaction between Cdc37 and the N-terminus of Hsp90, without binding to the ATP-pocket of Hsp90. (**A**) Chemical structure of elaiophylin. (**B**) Coimmunoprecipitation of Cdc37 with Hsp90 from MDA-MB-231 or MIA PaCa-2 cell lysates treated for 24 h with either 0.1% DMSO vehicle control, 1 μM elaiophylin, or 10 μM conglobatin A as indicated. Vehicle control treated cell lysate incubated with mouse IgG isotype control was used as a negative control; note the faint band in the Cdc37 probed line corresponds to the IgG molecular weight. (**C**,**D**) Elaiophylin was tested at increasing concentrations in a lysate-based split Renilla luciferase assay using NRL-Hsp90/ Cdc37-CRL (**C**) or NRL-N-Hsp90/ Cdc37-CRL (**D**). (**E**) Fluorescence polarization assay detecting competition of indicated compounds tested at 10 μM with fluorescently labelled geldanamycin for the ATP-pocket of Hsp90. (**F**) Computational docking of elaiophylin (cyan sticks) to the N-terminus of human Hsp90 (green; PDB ID 3T0Z). The interacting residues in Hsp90 are shown as gray sticks, ATP (magenta sticks) and magnesium (deep purple sphere) are indicated. The polar interactions between elaiophylin and the protein are shown as dashed lines with distances in Ångströms. The statistical significance levels are annotated in the plots as *** *p* < 0.001; **** *p* < 0.0001.

We recently described the mechanism of how the Hsp90/ Cdc37 interface inhibitor conglobatin A selectively targets K-Ras nanoclustering [15]. When performing a chemical similarity search using conglobatin A as a template, we identified elaiophylin as a related compound (Figure 1A). We therefore tested whether elaiophylin has a similar ability to disrupt the complex formation of Hsp90 and Cdc37. First, we detected the interaction between Hsp90 and Cdc37 in immunoprecipitation experiments by pulling down Cdc37 with a Hsp90α-specific antibody from MDA-MB-231 or MIA PaCa-2 cell lysates. Both elaiophylin and conglobatin A treatment reduced the amount of co-precipitated Cdc37 (Figure 1B).

We quantified this interaction and mapped it to the N-terminal domain of Hsp90, by employing two cell-lysate-based split Renilla luciferase assays [23]. In the first assay, the N-terminal fragment of Renilla luciferase was genetically fused to full-length Hsp90 (NRL-Hsp90) and then co-expressed with a fusion protein of the C-terminal fragment of Renilla luciferase and Cdc37 (Cdc37-CRL). This assay configuration can distinguish between ATP-pocket binders, such as 17-AAG, geldanamycin, or luminespib, which do not affect the luminescence signal, while Hsp90/ Cdc37 interaction disruptors decrease the signal [23]. Consistently, we observed IC_50_ (Hsp90/ Cdc37) = 14 ± 5 µM for elaiophylin (Figure 1C), which was 1.6 × more potent than conglobatin A (IC_50_ = 23 ± 6 µM, [15]). We pinpointed this activity to the N-terminus of Hsp90 using a second assay, which utilized a Renilla-fragment fusion with the N-terminal fragment of Hsp90 (NRL-N-Hsp90) [15]. Using this second configuration, we determined a similar micromolar inhibitory activity with an IC_50_ (N-Hsp90/ Cdc37) = 11 ± 4 µM (Figure 1D).

Next, we validated that elaiophylin does not act as an ATP-pocket competitor, using a fluorescence polarization assay that analyzed the competition with a fluorescent geldanamycin derivative [15]. While ATP-pocket binders 17-AAG, geldanamycin, and luminespib decreased the polarization signal, indicating binding to the ATP-pocket, elaiophylin did not, similarly to what we observed for conglobatin A (Figure 1E) [15].

In order to understand the structural basis of the compound binding to the target, we computationally docked elaiophylin to the structure of human cytoplasmic Hsp90α (gene HSP90AA1; N-Hsp90 in ATP-bound state, PDB ID 3T0Z), revealing a similar binding across a large surface area on Hsp90, as previously found for conglobatin A [15] (Figure 1F). Specifically, the computational docking of elaiophylin suggested H-bonding between elaiophylin and Ser50, Ser53, Asp54, Asp57, Ser129, Gln133, and His210, as well as hydrophobic interactions with Phe213. Thus, it appears that elaiophylin occupies important residues (Glu 47, Asp54, Gln133) that are otherwise bound by Cdc37 and sterically interferes with the Cdc37 binding.

These data suggest that elaiophylin is another potent protein–protein interface inhibitor of the complex of Cdc37 with the N-terminus of Hsp90, with no binding to the N-terminal ATP-pocket of Hsp90 and a similar binding mode to Hsp90 as conglobatin A.

### 3.2. Elaiophylin Affects Biomarkers Characteristic of a Hsp90/ Cdc37 Inhibitor and Acts K-Ras Selectively

A hallmark of Hsp90 inhibition is the loss of kinases that depend on the chaperone activity for their correct folding in order to avoid proteasomal degradation. In addition, Hsp90/Cdc37 interface inhibitors do not increase Hsp70 and Hsp90 levels, which are otherwise increased by ATP-pocket inhibitors due to the heat-shock response. We furthermore previously established that Gal3 levels decrease after Hsp90 inhibition, due to the loss of the transcriptional activator HIF1α, which is a Hsp90 client [15].

**Figure 2:**
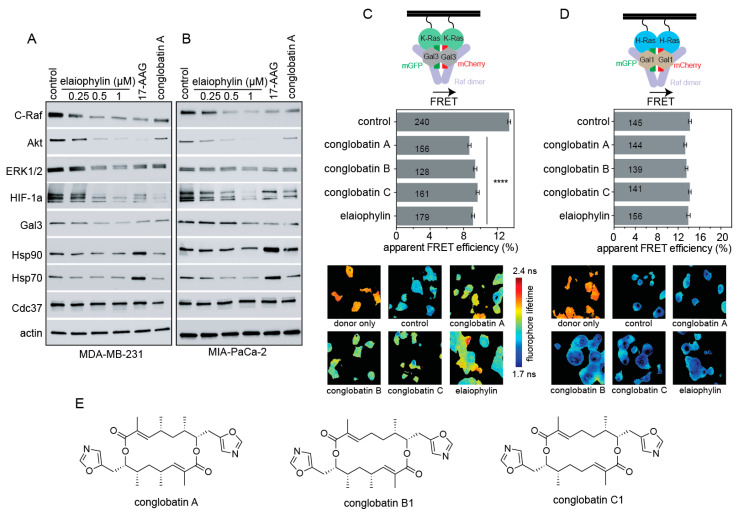
Elaiophylin treatment alters biomarkers of a Hsp90/ Cdc37 inhibitor and selectively inhibits K-Ras nanoclustering. (**A**,**B**) Western blots for indicated antigens of KRAS mutant MDA-MB-231 (**A**) and MIA PaCa-2 (**B**) treated with 0.1% DMSO vehicle control, 2 μM 17-AAG, 10 μM conglobatin A, or indicated concentrations of elaiophylin. (**C**,**D**) Ras nanoclustering measured by FRET in HEK cells co-expressing mGFP- or mCherry-tagged K-RasG12V (**C**) or mGFP- or mCherry-tagged H-RasG12V (**D**). Cells were treated with 0.1% DMSO control, 2 μM conglobatin A, conglobatin B1, conglobatin C1, or 1 μM elaiophylin for 24 h. Bottom, Examples of FLIM-FRET images of HEK cells with indicated treatments. “Donor only” refers to cells transfected with mGFP-RasG12V only (no FRET control). (**E**) Chemical structures of analyzed conglobatins. The statistical significance levels are annotated in the plots as **** *p* < 0.0001.

In agreement with these Hsp90/Cdc37 inhibitor characteristics, elaiophylin treatment of KRAS-G13D mutant MDA-MB-231 triple negative breast cancer cells (Figure 2A) or KRAS-G12C mutant MIA PaCa-2 pancreatic cancer cells (Figure 2B) led to a dose-dependent decrease of kinases C-Raf, Akt, and ERK1/2, while Hsp70 or Hsp90 were not induced. The latter contrasts to the typical induction of Hsp70 and Hsp90 observed with ATP-pocket inhibitors, such as 17-AAG (Figure 2A,B). Consistent with our previously elaborated mechanism, Hsp90 inhibition decreased HIF1α levels and concomitantly those of its transcriptional target Gal3 (Figure 2A,B). In agreement with the higher in vitro activity, elaiophylin showed notable effects already in the 1 µM range, while for conglobatin A we previously found around 10 µM to be required [15].

We then used our well-established Ras nanoclustering Förster resonance energy transfer (FRET) assay to monitor the effect of the compound on K-RasG12V and H-RasG12V nanoclustering [25]. In this assay, FRET fluorophores are genetically fused to the N-terminus of the Ras proteins. Dense packing of Ras in plasma membrane nanoclusters then allows for a high FRET signal, which can be abrogated if nanoclustering stabilizing components, such as Gal3, are lost [19]. In agreement with the observed loss of Gal3 upon elaiophylin treatment (Figure 2A,B), 1 µM elaiophylin selectively decreased the K-Ras-, but not H-Ras-nanoclustering FRET (Figure 2C,D). The activity of elaiophylin in this assay appeared comparable to that of conglobatin A, or two of its close derivatives conglobatin B1 and conglobatin C1 (Figure 2C–E).

Thus, elaiophylin treatment depleted several targets downstream of Hsp90, including HIF1α and its transcriptional target Gal3, which is a K-Ras-specific nanocluster scaffold. Consistently, elaiophylin selectively decreased the nanoclustering-dependent FRET of K-RasG12V, but not H-RasG12V.

### 3.3. Elaiophylin Inhibits Tumorosphere and Microtumor Growth

**Figure 3:**
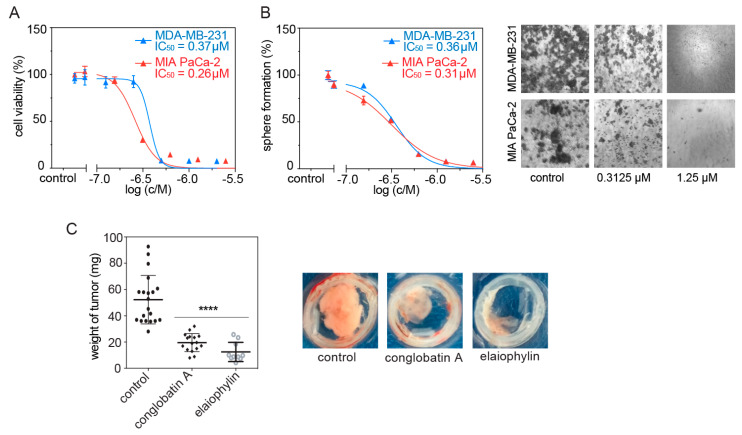
Elaiophylin potently reduces MDA-MB-231 3D spheroid growth and microtumor formation in the CAM model. (**A**,**B**) Dose–response analysis of elaiophylin tested for 72 h on MDA-MB-231 and MIA PaCa-2 cells in 2D cell proliferation (**A**) and 3D spheroid formation assays (**B**); *n* = 2. Right of (**B**) shows example images of spheroid formation after treatment with indicated elaiophylin concentrations. (**C**) Microtumor formation of MDA-MB-231 cells on the chick CAM. Cells growing into microtumors were treated for 5 days with 0.1% DMSO vehicle control, 10 μM conglobatin A, or 5 μM elaiophylin. Right, shows example images of microtumors. The statistical significance levels are annotated in the plots as **** *p* < 0.0001.

We previously showed that compounds, which selectively disrupt K-RasG12V, but not H-RasG12V nanoclustering, have the ability to block stemness properties of cancer cells, such as those assessed by mammosphere formation [13,25]. Consistently, we found that elaiophylin potently and dose-dependently decreased 2D cell proliferation (Figure 3A) and 3D mammosphere formation of MDA-MB-231 and MIA PaCa-2 cells (Figure 3B).

We next tested the effect of elaiophylin on the inhibition of microtumor growth of MDA-MB-231 cells when grown on the chorioallantoic membrane (CAM) of fertilized chick eggs. As compared to conglobatin A, elaiophylin reduced the size of microtumors significantly more (Figure 3C). These data confirm a strong anti-tumorigenic activity of elaiophylin.

## 4. Discussion

Here, we demonstrated that similarly to the related conglobatin A, elaiophylin inhibited the interaction between Hsp90 and its cochaperone Cdc37. In line with our previous mechanistic model for the K-Ras selectivity of conglobatin A, elaiophylin likewise decreased HIF1α levels and those of its downstream target Gal3. Given that Gal3 is a K-Ras specific nanocluster scaffold, loss of Gal3 explains the K-Ras-selective activity of elaiophylin treatment. Analogously to what we have recently published for conglobatin A, elaiophylin may have a high efficacy in KRAS mutant cancers with high HIF1α and Gal3 levels, which is particularly the case in pancreatic cancer [15]. In general cancer patients with this biomarker constellation have a worse survival prognosis [15]. In line with our proposed HIF1α—Gal3-associated mechanism for K-Ras selectivity, others have shown that elaiophylin decreases HIF1α expression in U87MG glioblastoma cells [33] and sensitized SKOV3 ovarian cancer cells to hypoxia [6].

Hsp90 is known to affect several steps of autophagy [34]. Autophagy is the process of controlled, lysosome-dependent degradation of cellular constituents, such as mitochondria (then also referred to as mitophagy). Autophagy is typically activated under various stresses and appears to have a mixed role in tumorigenesis, being generally protective against cancer, but then within a tumor it is exploited to fuel heightened energy demands [35]. In several tumor types, such as KRAS-mutant pancreatic and non-small-cell lung cancer, autophagy has emerged as a targetable process of interest [36,37]. Consistently, elaiophylin was found to inhibit autophagy and tumor growth in a SKOV3 ovarian cancer xenograft model [6]. In this context, it may be relevant that the stability of Ulk1, a Ser-Thr kinase required for mitophagy, depends on Hsp90/ Cdc37 [38]. In another example, ATP-pocket Hsp90 inhibitors blocked autophagy by promoting degradation of autophagy-associated proteins Atg7, Beclin-1, and Ulk1 [39,40]. In agreement with a particular significance of autophagy and Hsp90 inhibition, autophagy induction by the nonsteroidal anti-inflammatory drugs (NSAIDs), celecoxib and its COX-2-inactive derivative 2,5-dimethyl-celecoxib in CD44-high K562 cancer stem-like cells, sensitized these cells to Hsp90 inhibition with 17-AAG [41]. Of note, some NSAIDs are known to perturb the nanoscale organization of Ras proteins and their downstream signaling in the plasma membrane, which may point to a synergistic activity on Ras and autophagy in the above treatment combination [42].

## 5. Conclusions

Elaiophylin is another potent protein–protein interface inhibitor that interferes with the binding of Cdc37 to the N-terminal domain of Hsp90. Given the structural commonalities between the glycosylated polyketide elaiophylin and the previously investigated conglobatin A, we suggest that several other macrodiolides have a similar potential to inhibit the Hsp90/ Cdc37 interaction. Taken together with the in vivo data by others, one can tentatively conclude that Hsp90/ Cdc37 interface inhibitors have a low on-target toxicity. Given this profile, elaiophylin and related compounds may furthermore possess an interesting potential as novel autophagy inhibitors.

Our results furthermore confirm our recently proposed K-Ras-directed HIF1α and Gal3-dependent mechanism of action of Hsp90 inhibitors, suggesting that KRAS mutant cancers should be considered for the evaluation of Hsp90 inhibitors in the future. However, while the total synthesis of elaiophylin is established, high-affinity small-molecule Hsp90/Cdc37 interface inhibitors would have several benefits in terms of optimization of pharmacological properties, as well as optimization for high affinity to limit potential off-target effects.

## Data Availability

Not applicable.
